# Connexins—Therapeutic Targets in Cancers

**DOI:** 10.3390/ijms21239119

**Published:** 2020-11-30

**Authors:** Magdalena Nalewajska, Małgorzata Marchelek-Myśliwiec, Martyna Opara-Bajerowicz, Violetta Dziedziejko, Andrzej Pawlik

**Affiliations:** 1 Department of Nephrology, Transplantology and Internal Medicine, Pomeranian Medical University, Powstańców Wlkp 72 Street, 70-111 Szczecin, Poland; nalewajska@gmail.com (M.N.); malgorzata.marchelek@gmail.com (M.M.-M.); martyna.opara@wp.pl (M.O.-B.); 2 Department of Biochemistry and Medical Chemistry, Pomeranian Medical University, Powstańców Wlkp 72 Street, 70-111 Szczecin, Poland; viola@pum.edu.pl; 3 Department of Physiology, Pomeranian Medical University, Powstańców Wlkp 72 Street, 70-111 Szczecin, Poland

**Keywords:** connexin, hemichannel, gap junction, intercellular communication, cancer, cancer treatment

## Abstract

Connexins (Cx) are members of a protein family that forms intercellular channels localised in gap junction (GJ) plaques and single transmembrane channels called hemichannels. They participate in intercellular communication or communication between the intracellular and extracellular environments. Connexins affect cell homeostasis, growth and differentiation by enabling the exchange of metabolites or by interfering with various signalling pathways. Alterations in the functionality and the expression of connexins have been linked to the occurrence of many diseases. Connexins have been already linked to cancers, cardiac and brain disorders, chronic lung and kidney conditions and wound healing processes. Connexins have been shown either to suppress cancer tumour growth or to increase tumorigenicity by promoting cancer cell growth, migration and invasiveness. A better understanding of the complexity of cancer biology related to connexins and intercellular communication could result in the design of novel therapeutic strategies. The modulation of connexin expression may be an effective therapeutic approach in some types of cancers. Therefore, one important challenge is the search for mechanisms and new drugs, selectively modulating the expression of various connexin isoforms. We performed a systematic literature search up to February 2020 in the electronic databases PubMed and EMBASE. Our search terms were as follows: connexins, hemichannels, cancer and cancer treatment. This review aims to provide information about the role of connexins and gap junctions in cancer, as well as to discuss possible therapeutic options that are currently being studied.

## 1. Introduction

Connexins (Cx) are members of a protein family that forms intercellular channels localised in gap junction (GJ) plaques and single transmembrane channels called hemichannels [1,2]. Cx are expressed in almost every tissue of the human body (i.e., epithelial cells, smooth muscle cells, myoblasts, dendritic cells), except for red blood cells, spermatocytes and striated muscle cells [3,4]. In the majority of cases, each tissue type expresses more than one connexin, therefore creating an extensive spectrum of intercellular communication due to the possibility of heteromeric channels formed by compatible Cx [4,5,6,7,8]. In humans, 20 connexin isoforms have been described. The connexin family shows high similarity in terms of the amino acid composition as well as in the transmembrane domains. Based on the differences and similarities in the structure of individual connexins, they have been divided into subfamilies. Currently, five sub-families are described: GJA, GJB, GJC, GJD and GJE. This division is based on the structural similarity of genes, their homology and sequence as well as the length of the connexin cytoplasmic domain [4].

Connexins occur both in the form of cell-to-cell plasma membrane domains known as gap junctions (GJ) and single-membrane hemichannels [1]. Gap junction intercellular communication (GIJC) is responsible for the direct communication and exchange of small cytosolic molecules between contacting cells in a relatively non-selective manner. Therefore, GJs are involved in various physiological functions—i.e., the maintenance of cell homeostasis, growth and cell differentiation [9], glandular secretion [10], angiogenesis [11], neuronal migration [12] and stem cell development [13]. Hemichannels, however, remain free and unopposed, and their physiological role is to exchange molecules between the cytosol and the extracellular space [14,15,16]. Hemichannels play a crucial role in autocrine and paracrine signalling pathways [17].

The expression and physiological functions of connexins are well-documented, and the main challenge regarding connexins and gap junctions in general concerns both their roles in the pathophysiological mechanisms of various diseases and as novel interventional targets. Connexins have been already linked to cancers, cardiac and brain disorders, chronic lung and kidney conditions and wound healing processes. In cancers, connexins may be involved in alteration of intracellular communication, can interfere with signalling pathways or modulate cells by autocrine and paracrine mechanisms.

This review provides an overview of the role of Cx in cancer pathogenesis and progression and as possible targets for cancer treatment.

## 2. Structure and Life Cycle of Connexin Proteins

Connexin proteins form hexagonal structures termed connexons or hemichannels in the plasma membrane [18]. Connexons of contacting cells bind together to form the gap junction—i.e., a hydrophilic pore that enables direct cytosol-to-cytosol communication and the passage of molecules between neighbouring cells [18]. This docking process is supported by the presence of different adhesion proteins. Connexons consist of connexins, either the same or different, which create homomeric or heteromeric hemichannels, respectively.

Depending on the composition of connexons, intercellular channels of gap junctions could be classified as one of four classes: (1) homomeric-homotypic, which consist of two identical connexons formed by only one type of connexin; (2) homomeric-heterotypic, built from different connexons, each formed of the same type of protein; (3) heteromeric-homotypic of two identical connexons with at least two isoforms of connexins; (4) heteromeric-heterotypic, built from different connexons, each formed with two or more isoforms of connexins [18,19,20,21]. The type of gap junction has an impact on its biophysical properties [22,23,24].

All connexins share similar topology: they consist of four hydrophobic transmembrane domains (TM1-TM4) connected by two extracellular loops (E1-E2) and one cytoplasmic loop (CL). Moreover, connexins contain cytoplasmic C- and N-terminal regions [18,21,25]. The extracellular loops are responsible for docking processes [19,26,27,28]. The N-terminus participates in the oligomerisation of connexins and connexin trafficking [29]. It also produces a selectivity signal that allows selective interactions and the docking of connexin proteins [18,22]. The C-terminus plays a role in the phosphorylation of connexins: Cx31, Cx32, Cx37, Cx40, Cx43, Cx45, Cx47 and Cx50 [30,31,32]. The C-terminus also takes part in oligomerisation processes and affects the chemical and electrical conductivity of gap junction channels [18,30,33]. It has been shown that the C-terminus can also regulate intercellular Ca^2+^ flow by binding calmodulin [34]. Moreover, the C-terminus and CL are binding sites for different structural proteins—i.e., zonulin 1 and 2, claudin 1, as well as protein components of adherens junctions [35,36,37]. This enables the formation of an advanced complex of proteins resulting in increased stabilisation and regulated intercellular communication via signalling pathways [35,36].

Five decades after their discovery, the expression and physiological functions of Cx are well-documented. The main challenge regarding Cx and GJs in general concerns their role in the pathophysiological mechanisms of various diseases and as novel interventional targets. Cx were linked to cancers since shortly after their discovery, and it was hypothesised that altered gap junctional communication promotes cancer incidence and tumour proliferation [9,38,39]. A study by Loewenstein and Kanno was the first to reveal the absence of electrical coupling in rat liver tumour cells in comparison to healthy hepatocytes [40]. This phenomenon was later confirmed in human thyroid cancer [41] and cultured mammalian cancer cells [42]. Since then, knowledge of the role of Cx in cancer biology has widely expanded. It is now an established fact that Cx display various functions depending on the cancer type and disease stage, as well as on their isoform [9,43]. The underlying mechanisms encompass their roles in cell-to-cell communication as GJ components or, independent of GJs, as modulators of heterogeneous signalling pathways, including autocrine/paracrine pathways [44]. The complexity of Cx involvement in cancer biology provides a vast field of research for the use of Cx-targeting drugs as novel therapeutic strategies in cancer management.

## 3. The Dual Role of Connexins in Cancers

### 3.1. Connexins as Tumour Suppressors

In vivo and in vitro observations have indicated a role for GJs in the process of tumorigenesis. Data presented in recent decades have shown that GJIC is generally lost in rapidly dividing cells to prevent the exchange of essential metabolites between those cells and their non-dividing neighbours [45,46]. GJIC depletion has been widely observed in solid tumours, confirming the negative correlation between connexin expression and tumour growth [9]. This observation served as the basis for the hypothesis that Cx could be tumour suppressors. This role has been confirmed in various studies performed in knock-out (KO) models. For instance, Cx32 KO mice are more susceptible to spontaneous [47] or chemically-induced liver tumours [48], and these results were compared with previously published data on liver tumours in rodents and humans [49,50]. In these studies, GJIC was lost due to the absence of Cx32 expression or its ectopic cytoplasmic localisation. Moreover, in Cx32 KO mice, the formation of multiple tumour types was augmented following irradiation by X-rays [51]. In a recent study, overexpression of Cx43 demonstrated an antimetastatic effect on MDA-MB-231 breast cancer cells in vitro and in vivo and in human breast cancer tissues [52]. Cx32 is also responsible for the inhibition of hepatocellular carcinoma invasion and metastasis [53]. The restoration of GJIC function generally results in a decrease in tumour growth, as demonstrated by the use of chemopreventive agents (i.e., flavonoids or carotenoids) or cDNA transfection [44,54,55,56]. The effect, however, is dependent on the transfected Cx subtype [57].

Unfortunately, it is not clear how Cx modulate cell growth. Studies on various cancer cell lines led to the conclusion that overexpression of Cx is linked to an elongation of the G1 phase of the cell cycle, resulting in decreased cell proliferation [44,58]. The mechanism that underlies G1/S cycle arrest is related to the accumulation of p27 [59]. Elevated expression of p27 follows the flux of its enhancer, the cyclic 3′,5′-adenosine monophosphate (cAMP), via the GJ-dependent pathway [59]. Additionally, p27 levels increase due to the degradation of S-phase kinase-associated protein 2 (Skp2), a protein that is responsible for p27 ubiquitination and degradation, which is driven by Cx43 itself [59,60]. Cx43 increases the instability of Skp2 protein and suppresses its expression [59,60]. The connexin-derived control of nuclear processes occurs through the inhibition or activation of other cell cycle regulators. Cx43, as a single entity, promotes the accumulation of p27, inhibits the activity of cyclin-dependent kinases (CDK), cyclin D1 and Skp2 and acts as a sequestrator of various growth modulators. Among them are CCN3, overexpressed in nephroblastoma, PTEN (phosphatase and tensin homolog), C-terminal Src kinase (Csk) or proto-oncogene tyrosine-protein kinase (c-Src) [44].

The specific permeability of various Cx plays a role in GJIC-mediated growth control mechanisms. In an osteoblastic model, the transition of second messengers through GJ leads to the activation of ERK and PI3/Akt pathways. The cAMP Cx-mediated redistribution between contacting cells results in prolongation of the G2/M cell cycle phase. Other molecules can also pass through GJs—e.g., microRNAs (miRNAs) [44,61].

Cx can also control tumour growth through channel-independent mechanisms. The cytoplasmic domains of Cx, especially the C-terminus, can interfere with other cytosolic/membrane proteins [44]. In this setting, they can modulate cell growth by adjusting channel permeability, and by interfering with plasma membrane-nucleus signalling pathways. Phosphorylation of the Y247, Y265 and Y313 residues on the Cx43 CT domain in a Src-related manner causes channel closure [62,63]. In addition to broad cytoplasmic localisation, CT-Cx43 also localises to the nucleus. It is, therefore, suggested that nuclear localisation of CT-Cx43 may exert effects on gene expression and growth [64]. As for the second mechanism, CT regions can modulate the activity of their protein partners. Such a correlation has been described for Skp-2 and Cx50 [65], β-catenin and Cx43 [66], discs large homologue 1 (Dlgh1) and Cx32 [67] and Cx43 [68]. Cx32 and Dlgh1 together play a role in cancer progression in an oncoprotein E6-related manner [17,67]. Interaction of Cx43 with β-catenin in the nucleus downregulates genes involved in metastasis [17,66]. Similar mechanisms occur for other structural proteins—i.e., ZO-1 [17,69]. Another tumour suppressive role of CT domains has been described recently, as the 266–283 region in the CT domain of Cx43 binds to c-Src and its inhibitors CKS and PTEN, inhibiting the oncogenic activity of c-Src [70]. Hemichannels are also linked to the modulation of cell-proliferation; however, the exact role of this is yet to be elucidated [71].

As explained above, decreased Cx expression or loss of function is commonly observed in cancer. At the transcriptional level, connexin expression is lowered due to alterations in the activity of transcription factors and epigenetic control of connexin mRNA [9,17]. Epigenetic silencing is mainly related to histone acetylation and the activity of histone acetyltransferase and deacetylase that promote and inhibit transcription, respectively, and to promoter hypermethylation by DNA methyltransferase [72]. Methylation of connexin genes has been linked to the loss of Cx43 expression in HeLa cells [73] and small-cell lung cancer [74], Cx32 in renal cell carcinoma [75] and Cx45 in colon cancer [72]. Another mechanism includes the post-transcriptional modification of connexin mRNA by cancer-related microRNAs. In the literature, the inhibitory effects of the mi-R-221/222 complex and miR-125b on Cx43 have been described in glioma [76] and that of miR-20a in prostate cancer [77]. Furthermore, alterations in the synthesis of truncated forms of Cx—i.e., GJA1-20k—in Smad3/ERM-related pathways [17] are believed to play a role of chaperone proteins, enabling the trafficking of regular Cx to the cell wall, with their synthesis being mainly moderated by mTOR and Mnk1/2 signalling [32,78,79]. Post-translational modifications include phosphorylation, acetylation, ubiquitination and SUMOylation, with phosphorylation being the most widely studied process in cancers [80]. Various kinases and phosphatases—i.e., MAPK (mitogen-activated protein kinase), PKC and PKA (protein C and A kinases) and cdc2/cyclin B—modulate Cx43 phosphorylation, leading to the inhibition of GJIC [9,17]. Phosphorylation interacts with connexin trafficking, stabilisation of the cell membrane and protein-to-protein communication [17,81,82].

Finally, some studies have been performed on the coding regions of Cx genes or their promoters [44]. However, in contrast to classical suppressors—i.e., p53 and Rb—mutations in Cx genes are rare. One example can be found in the literature: in colon adenocarcinoma, mutations have been documented in the CT region of Cx43. Those mutations led to a shift in the reading frame for all cases and were only related to invasive tumours [83]. Nonetheless, further studies are required to verify this hypothesis.

### 3.2. Connexins as Promoters of Cancer Progression and Metastasis

Recent studies suggest that connexin expression may promote tumour malignancy under certain conditions [17]. Some Cx act as promoters of invasion and metastasis, especially in advanced stages of cancer. In some cancer cells, restoration of Cx promotes migration and invasion [84,85], intravasation and extravasation [86,87], metastatic growth [88,89] and resistance to therapy [17].

Increased expression of Cx43 and its membrane localisation has been observed in various cancer types [90]. Cx43-composed GJs between cancer cells and astrocytes have been linked to increased growth of brain metastases through cGAMP signalling [91]. Cx43 levels are increased in metastatic lymph nodes compared to primary site tumours [92]. Moreover, the levels of Cx43 miRNA are also increased in metastases [93], and metastatic tumours display Cx43 positivity, even when primary breast tumours are Cx43 negative [92]. The same observations have been made for Cx26 [90]. Metastatic breast cancer cells have been shown to display increased levels of Cx26 compared to primary tumours, and surface Cx26 has been observed only in metastatic cells [92]. In colorectal cancer, lung metastases also display increased levels of Cx26 compared to primary tumours [94]. Furthermore, Cx26-GJIC was noted to participate in cancer cell migration in a HeLa cell model [95] in which HeLa cells overexpressed Cx26, enabling single-cell migration [95]. Therefore, it was concluded that migration might occur due to the inhibition of cell–cell adhesion. Chandrasekhar et al. showed that PKA is necessary for Cx26-induced tumour growth inhibition [96]. Activation of PKA was associated with a redistribution of cAMP in the cells, but Cx43 and Cx32 failed to mediate this redistribution. In a study conducted by Chen et al. on mice, it was found that protocahedrin is strongly associated with breast and lung cancer cells, which connects them with astrocytes in the brain and through Cx43-GJ enables the transfer of second messenger cGAMP to astrocytes. As a result, metastatic cells are capable of growth and become resistant to chemotherapy [91]. Studies performed in recent years suggest that selected connexins—i.e., Cx43, Cx26 and Cx32—could serve as biomarkers in prognosing the course of cancers or exploited for therapeutic strategies in cancer management [90].

GJIC between cancer and endothelial cells is also linked to cancer progression [97]. This concept was first introduced for melanoma [86]. BL6 cells expressing Cx26 were shown to form coupling with endothelial cells, in contrast to Cx26-deprived F10 cells. BL6 subclones were associated with a higher metastatic potential than F10 subclones. Expression of Cx26 was low in melanoma cells that were located in the basal layer and considerably upregulated in invading cells. The authors concluded that Cx26 might be involved in the intra- and extravasation of melanoma cells due to the formation of GJ with endothelium. This conclusion was later verified in breast cancer and brain metastases [98]. Breast cancer cells formed functional GJ with brain endothelium in a Cx43-related manner. Upregulation of Cx43 was crucial for extravasation and co-opting blood vessels. Concordant results were obtained in a study on malignant glioma cells, which formed Cx43-mediated GJIC with neighbouring astrocytes, leading to the promotion of tumour invasion [99]. However, in a recently published report, hypoxia-induced internalisation and degradation of Cx43 and Cx26 were linked to increased proliferation and migration of non-small lung cancer cells [100].

The term “cancer stem cells (CSCs)” accounts for the subpopulation of cancer cells that present pro-tumorigenic properties [101,102]. Some authors even suggest that CSCs play a crucial role in tumour occurrence, progression and metastasis, as the introduction of CSCs into healthy subjects results in the development of specific tumours [102]. CSCs have been found to contain Cx [102]. However, the effect of Cx on CSCs depends both on their membrane and cytoplasmic localisation. In cancer, Cx participate in intercellular communication in the form of hemichannels and GJs, or act as cytoplasmic proteins through kinases and transcription factors [102]. The role of Cx in breast cancer stem cells (BCSC) has been well-documented [17,102]. In triple-negative breast cancer, upregulation of Cx26 has been observed. However, the pro-tumorigenic effect of Cx26-mediated BCSC relied upon its connection to focal adhesion kinase (FAK) and the transcription factor NANOG, instead of its channel-forming properties. The mentioned complex was found to interact outside the nucleus to phosphorylate NANOG [103]. In glioma stem cells (GSCs), higher levels of Cx46 wwere identified in comparison to regular cancer cells, and this correlated with proliferation and self-renewal [104]. In contrast to Cx26 in breast cancer, the role of Cx46 in GSCs is membrane- and GJIC-related [102,104]. In another study, decreased levels of Cx43 were described for GSCs [105] and related to increased capacity for self-renewal, proliferation and tumour formation [105]. Moreover, Cx43 restoration resulted in a reversal of these effects [105]. In a study on hepatocellular carcinoma cells, the accumulation of cytoplasmic Cx32 promoted tumour progression and metastasis via cancer stem cell self-renewal [106]. Table 1 shows clinical relevance of connexins in some types of cancer.

**Figure 1:**
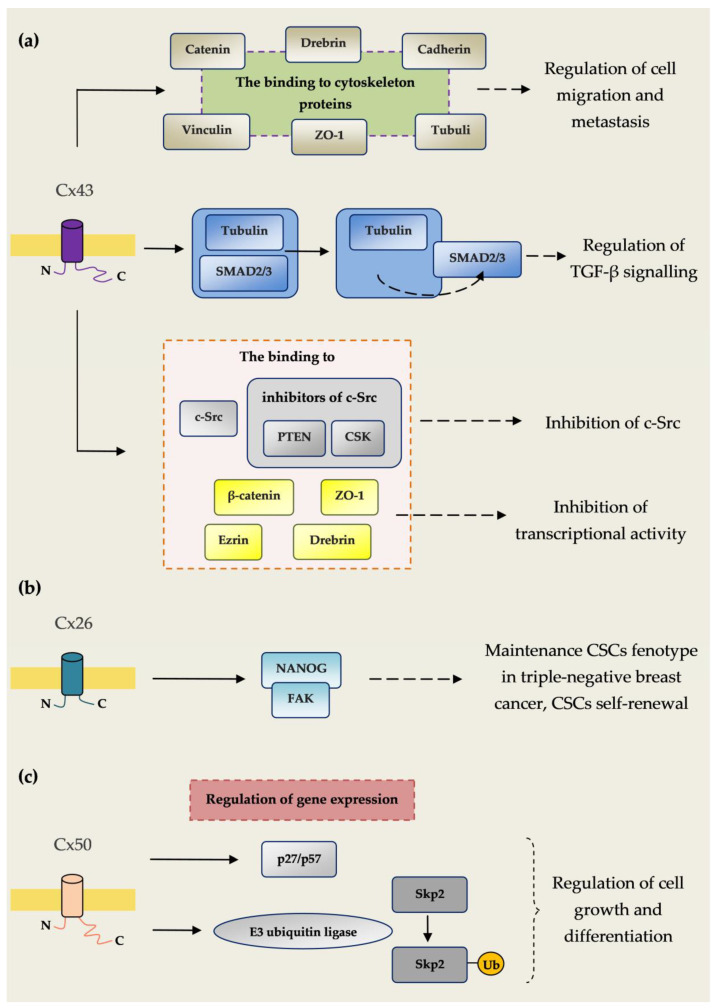
Connexin–protein interactions influencing carcinogenesis. (**a**) The binding of Cx43 to cytoskeleton proteins tubulin, cadherins, catenins, vinculin, ZO-1 and drebrin regulates cell migration and metastasis. Cx43 inhibits the connection of Smad2/3 with tubulin, causing the secretion of Smad2/3, which regulates pathways associated with TGF-β. TGF-β signalling plays an important role in many cancers such breast, colon, lung, pancreatic and prostate cancer. Cx43 enhances c-Src blockade, and by a connection with c-Src as well as CSK and PTEN, which are c-Src endogenous inhibitors. C-Src tyrosine kinase is a proto-oncogene involved in many cellular pathways such as cell migration, proliferation and survival. The dysregulation of c-Src leads to malignant transformation and has been observed in several cancer types. C-Src tyrosine kinase also plays an important role in resistance to chemotherapy. Cx43 inhibits in the nucleus the transcriptional activity of β-catenin, drebrin, ezrin and ZO-1 regulating the expression of genes controlling the process of carcinogenesis. (**b**) Cx26 plays an important role in maintenance of the cancer stem cell (CSC) phenotype in triple-negative breast cancer. Cx26 enhances CSC self-renewal by interaction with the pluripotency transcription factor NANOG and focal adhesion kinase (FAK). (**c**) Cx50 regulates the expression of the cyclin-dependent kinase inhibitor p27/p57 and E3 ubiquitin ligase Skp2. Cx50 enhances auto-ubiquitination and subsequent degradation of Skp2. Through this mechanism, Cx50 regulates the expression of mediators regulating cell growth and differentiation [17].

Figure 1 Shows connexin–protein interactions influencing carcinogenesis.

### 3.3. Role of Connexins in Chemo- and Radiotherapy

#### 3.3.1. Resistance to Chemotherapy

Cx-related resistance to anti-cancer treatment has been recently reported [17]. Cancer cells could be resistant to radio- or chemotherapy through GJIC-dependent and independent mechanisms [17,118]. In a study on glioma cells [119], the protective role of neighbouring astrocytes was described in relation to chemoresistance. The protective effect was demonstrated following treatment with temozolomide, cisplatin and fluorouracil. The authors emphasised that the chemoprotective effects of astrocytes relied upon direct contact between astrocytes and glioma cells and was GJ-related. Cx43 was shown to play a crucial role in this phenomenon. A similar observation was made for melanoma brain metastases [120]. The authors revealed that astrocyte-related chemoprotection occurred through Cx43-GJ [120]. Sequestration of Ca^2+^ has been listed as the most probable mechanism by which astrocytes display their protective function on cancer cells [120]. In another study, breast and lung cancer cells have been shown to establish GJs from Cx43 and protocadherin 7 with astrocytes in the brain [91]. This has been linked to the transfer of cGAMP from cancer cells to astrocytes and the release of inflammatory molecules interferon α (INFα) and tumour necrosis factor α (TNFα) via the STING pathway, resulting in increased tumour growth and chemoresistance [91]. A study of lung cancer cells and Cx30.3 (GJB4) revealed the increased metastatic potential and enhanced chemoresistance towards gemcitabine and etoposide in a Src-related manner [121]. Interestingly, pro-oncogenic properties occurred despite the absence of GJs, indicating the presence of GJIC-independent mechanisms of chemoresistance [121]. Another study of non-small lung cancer revealed a correlation between resistance towards gefitinib and the upregulation of Cx26 [122]. However, chemoresistance occurred via a GJIC-independent pathway and was an outcome of increased PI3K/Akt phosphorylation [122]. The upregulation of Cx32 has also been linked to increased cisplatin resistance in human ovarian cancer cells via the modulation of drug efflux transporters and activation of the EGFR-protein kinase B signalling pathway [123]. The same Cx has been shown to facilitate hepatocellular carcinoma progression by increasing chemoresistance via modulating Src and favouring EGFR phosphorylation [89].

#### 3.3.2. Resistance to Radiotherapy

In a study performed in 1981 [124], a correlation was detected between GJIC and radioresistance in spheroids. In 2013, different responses of human cells to ionising radiation were observed based on the type of Cx involved [125]. Irradiated HeLa cells expressing Cx32, but not Cx26, showed an increased radioresistance rate compared to controls. It has not been ultimately settled whether these effects depend on GJIC or not. Some authors suggest that the opposing effect of irradiation on HeLa cells expressing different Cx could depend upon differential interactions with other proteins—i.e., cytoskeletal proteins, β-catenin, kinases, as well as with molecules that communicate through different GJs. Moreover, the effect could also depend upon the character of the ionising radiation—i.e., in relation to LET (linear energy transfer). High-LED particles—i.e., iron ions or α-particles—display more chemically active species than low-LED particles, and iron ions also generate secondary radiation that is assumed to interact with signalling pathways that are Cx-mediated. However, the authors pointed out that studies using lower doses of irradiation should be performed to further confirm this thesis. In another study on Cx30 and malignant astrocytic gliomas, the suppressive effect of the studied Cx molecules was confirmed in relation to tumour growth [126]. However, in the same study, Cx30 was shown to display a protective effect on glioblastoma cells in terms of radiation-mediated DNA damage [126]. These radioprotective properties were due to the translocation of mitochondrial heat shock protein 90 and increased production of ATP [126]. In a study by Osswald et al., it was confirmed that glioblastoma cells that established functional Cx43-mediated GJs displayed decreased levels of intracellular Ca^2+^ and were less sensitive towards radiation compared to unconnected cells [127].

#### 3.3.3. The Bystander Effect

Discovery of X-rays and ionising radiation was followed by its application in inducing proliferating cancer cell damage due to the generation of single- and double-stranded DNA breaks [118]. In recent years, it has been revealed that ionising radiation destroys not only targeted cells but also cells that have not been directly irradiated [128,129]. This so-called ”bystander effect” is assumed to occur thanks to signal transduction via GJs [129]. This phenomenon has been described in various studies [130]. The bystander effect plays a role in resistance mechanisms to radiotherapy with a positive correlation between GJIC and radiotherapy resistance confirmed in cultured multi-cell spheroids [124]. In another study, the restoration of Cx30 resulted in decreased radiation-induced mortality and DNA damage in GBM cells [126]. Some authors list other factors besides irradiation that could induce the bystander effect [17,118]. Reactive oxygen species, reactive nitrogen species, Ca^2+^, protein factors and various cytokines can spread via GJ to neighbouring cells to induce damage [17,128,131]. The concept of the bystander effect could result in newer therapeutic strategies, as understanding of the underlying biology (blockage or restoration of GJ) could produce either a protective effect on surrounding cells or enhance damage to malignant cells [17,118].

## 4. Therapeutic Strategies Involving Connexins

The complex role of Cx and GJs in cancer biology provides a broad spectrum of possible therapeutic strategies. A few approaches have been proposed in recent decades; however, further exploration is required to establish their potential. Downregulation of Cx or GJIC has been commonly observed in cancer cells, especially in early-stage cancers. Restoration of GJIC and increased Cx expression has been proposed by some as a possible therapeutic approach [17]. Many natural-based or synthetic chemical compounds exhibit the potential to modify Cx and/or GJIC [17,44].

Retinoids, vitamin D, carotenoids, flavonoids, green tea catechins, red wine resveratrol or statins are among the molecules that could reverse GJIC/Cx deficiency [17,44]. For instance, lycopene, one of the carotenoids present in many fruits and vegetables, was found to inhibit cell proliferation in the breast tumour cell line MCF-7 by stimulating GJIC functionality and increasing Cx43 expression [132]. High levels of mevalonate have been observed in various cancers, so inhibition of the mevalonate producer β-hydroxy-β-methylglutaryl-CoA (HMG-CoA) reductase could result in cancer regression [133]. In a study of transformed E9 mouse lung cells, lovastatin increased GJIC but not Cx43 expression in cancer cells through the inhibition of protein kinase C [134]. In a study on simvastatin, its augmentative effect on GJs composed of Cx43 was revealed in Leydig tumour cells [135]. In the same study, upregulation of GJs by simvastatin sensitised cancer cells to the chemotherapeutic drug etoposide [135]. On the contrary, upregulation of Cx43 and GJIC by simvastatin attenuated the toxicity of cisplatin on normal Sertoli cells [136].

Chemotherapeutic agents also display modulating potential towards Cx or GJIC expression [137]. Fukushima et al. showed that therapy with a combination of a Cx43-expressing plasmid and docetaxel enhanced tumour suppression in human prostate cancer cell cultures by increasing the activity of apoptotic molecules and downregulating Bcl-2 expression [138]. This study was the first to indicate the beneficial effect of combined therapy. In 2009, Sato et al. described augmentation of carboplatin-induced toxicity in the mesothelioma cell line H28 via the upregulation of Cx43 [139]. The underlying mechanism depended on Cx-related suppression of c-Src [139]. Cx43 has also been able to enhance sunitinib toxicity in H28 cells through activation of Bax and phosphorylation of c-Jun N-terminal kinase [140]. Eicosapentaenoic acids have been described to promote chemosensitivity towards to 5-fluorouracil in melanoma models by increasing Cx43 expression [141]

Other therapeutic approaches embrace the blockage of GJIC and Cx by various molecules, as Cx facilitate tumour growth and metastasis in some cases [9,17]. Oleamide, an organic amide derived from oleic acid, has an inhibitory effect on pulmonary and hepatic metastases due to the modulation of cancer cell extravasation; this was demonstrated using MDA-MB-231 human breast cancer cells in vitro and a xenograft murine model in vivo. [142]. In a study by Chen et al., both tonabersat and meclofenamate displayed inhibitory potential on brain metastases from lung and breast cancer in mouse models by blocking GJs between cancer cells and astrocytes [91]. Addition of either compound to carboplatin therapy further augmented the therapeutic properties [91]. A clinical trial concerning the effect of meclofenamate treatment in recurrent or progressive brain metastases from solid tumours is currently being conducted in humans (NCT02429570). In a bone metastasis model, Ca^2+^ signalling from osteogenic cells to cancer cells through GJs played a pivotal role in the formation of bone metastases, alongside the mTOR signalling pathway. Blockage of calcium flow between cells by GJ inhibitors (carbenoxolone, arsenic trioxide) reduced the progression of bone metastases in mice, implicating these agents as potential therapeutic candidates [112,143]. Carbon monoxide (CO) is another compound that could inhibit hemichannels in the treatment of cancer [44]. It has been observed that CO donors (CORM-2) display inhibitory potential on Cx46 hemichannels in *Xenopus laevis* oocytes in a dose-related manner [144,145,146]. The same observation has been made for Cx46 and Cx43 hemichannels in HeLa cells [144,145,146]. However, the exact mechanism is yet to be fully determined.

Another promising strategy concerns the usage of connexin mimetic peptides and specific antibodies to regulate GJ and connexin expression [9,17,147]. This strategy exceeds that which was previously described, as it is more specific and may reduce the number and severity of adverse effects [9,17]. A αCT1- the Cx43-C-terminal mimicking peptide has been shown to block the ZO-1 interference with Cx43 sensitised temozolomide-resistant human glioblastoma cells to temozolomide treatment [148]. Moreover, the combination of the Cx43 hemichannel inhibitor and temozolomide resulted in an augmented therapeutic effect [148]. In another study, targeting Cx40 with a mimetic peptide decreased vascularisation and tumour growth in mice [149]. In a different context, the mimicking peptides also enhanced GJIC. αCT1 application enhanced GJ activity and reduced tumour proliferation in breast cancer cell lines [150]. Addition of αCT1 to tamoxifen for the treatment of ER-positive and to lapatinib for the management of HER2-positive breast cancer cells led to more positive effects [150]. In another study, the cell-penetrating Cx-based peptide TAT-Cx43_266-283_ displayed anti-tumour properties in human glioblastoma models by reducing migration and the invasive properties of glioblastoma stem cells [151]. Moreover, monoclonal anti-Cx43 antibodies have been successfully used to reduce tumour growth as monotherapy [152] and in combination with standard chemo- and radiotherapy [153]. In a recently published study, the role of Cx43 and GJIC in melanoma cell killing by cytotoxic T lymphocytes was demonstrated [154]. The authors suggested possible immune therapeutic strategies in the treatment of selected cancers based on Cx43 levels [154].

**Figure 2:**
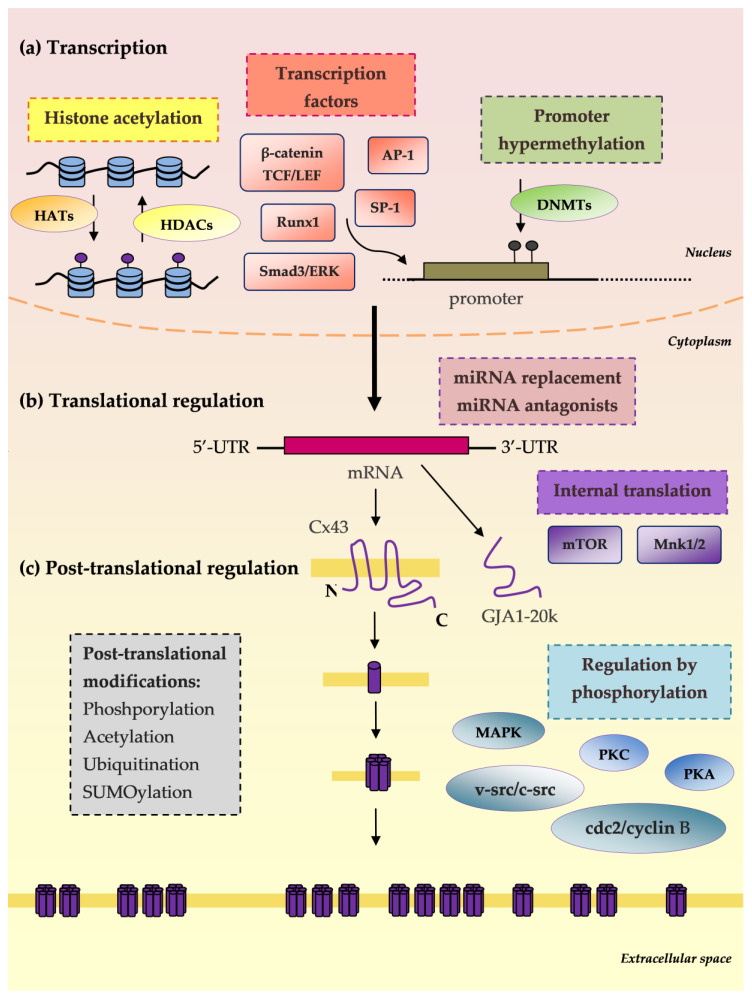
Connexin synthesis steps as therapeutic targets. (**a**) Transcription: Histone acetylation: histone acetyltransferase enzymes (HATs), histone deacetylases (HDACs); Transcription factors: Runx1, Ap-1, Sp-1, β-catenin, TCF/LEF; Promoter hypermethylation: DNA methyltransferase enzymes (DNMTs). (**b**) mRNA translational regulation: microRNA replacement; microRNA antagonists; Internal translation: mTOR and Mnk1/2; GJA1-20k (truncated forms of Cx43): Smad3/ERK-dependent repression of GJA1-20k reduces Cx43 gap junctions. (**c**) Post-translational regulation: Phosphorylation: mitogen-activated protein kinase (MAPK), protein kinase C (PKC), protein kinase A (PKA), cdc2/cyclin B and v-Src/c-Src; Acetylation; Ubiquitination; SUMOylation [17].

Most of the abovementioned therapeutic strategies are not directional towards Cx and GJIC and, therefore, are responsible for a number of side effects depending on the receptors they interact with. Currently, effort should be placed on targeted drug delivery to the tumour location or its surroundings in order to minimise deleterious interactions with healthy cells. Connexin targeting nanocarriers are a promising approach. In a study by Baklaushev et al., cisplatin-loaded nanogels were conjugated with monoclonal antibodies to Cx and BSAT1 for the treatment of rat glioma [155]. The nanogels induced a significant decrease in tumour growth, most probably due to antibody-mediated adhesion of cisplatin-loaded nanogels to the tumour site and their more efficient internalisation. In a study by Chekhonin et al., PEGylated immunoliposomal nanocontainers combined with monoclonal antibodies against the second extracellular loop of Cx43 have been used in the therapy of malignant intracranial C6 gliomas in mice [156]. Table 2 summarises known approaches to target connexins and gap junctions in the treatment of cancer with chemical modulators—i.e., enhancers and inhibitors, as well as connexin-specific targeted drugs. Figure 2 shows connexin synthesis steps as therapeutic targets.

## 5. Conclusions and Future Perspectives

Gap junctions and hemichannels formed by connexins are responsible for cell–cell communication, the passage of small molecules between contacting cells and between cells and the extracellular milieu. They can also display activities independently of GJIC, thus impacting the maintenance of cell homeostasis. The complexity of Cx biology has been a foundation for exploring the role of Cx and GIJC in the onset of various diseases, including cancer. The exact role of Cx in cancer initiation and development is yet to be elucidated. To date, it has been established that Cx can either promote or suppress tumour growth, depending on the connexin isoform or cancer stage/type. As a result, investigations have focused on tissue-specific roles of specific Cx in relation to cancer. In various studies, utilisation of connexin modulators as a therapeutic option in cancer management is currently being verified. However, connexin modulators affect not only cancer cells, resulting in cancer depletion, but also normal, non-cancerogenic cells, thereby producing serious side-effects. There is a need for further development of specific targets toward cancer-related Cx, as well as combination treatment with standard therapeutics.

The effect of connexins on tumour growth depends on the connexin isoform, the stage of the cancer and the type of tissue. Previous studies have shown anti-tumorigenic properties of connexins and reduced connexin expression in tumours, especially Cx43, Cx26 and Cx32 [59,100,159,160,161,162]. Reduced expression of connexins was found mainly in breast and lung tumours, but also in other types of cancers. It has been shown that an experimental increase in connexin expression in cancer cells decreases tumour growth, while the inhibition of connexin expression enhances the growth and invasion of cancer cells [59,163,164,165]. However, some studies have shown that connexins may also exert pro-tumorigenic effects by increasing the migration and invasion of cancer cells and may increase chemoresistance [88,89,123,166]. In prostate cancer cells, high expression of Cx43 was correlated with a more aggressive phenotype and a high incidence of osteolytic metastases [89]. Overexpression of Cx32 in hepatocellular carcinoma was associated with advanced stage, poor tumour differentiation and worse prognosis [166]. Cisplatin resistance in women with ovarian cancer was correlated with the upregulation of Cx32 expression [123].

Identifying mechanisms by which different connexins impact different stages of cancer is of particular interest and needs further verification. The exact role of channel-dependent and independent pathways in cancer cell growth and differentiation also requires elucidation in the context of targeted treatment. The contribution of pannexins, a family of proteins showing similar properties to connexin hemichannels, should also be taken into consideration. However, the determination of connexin and pannexin function could remain a challenge. The recognition of novel and tumour-specific connexin modulators that would minimise the adverse effects of blocking or enhancing GJ in other tissues is another issue of great importance.

The multidirectional involvement of connexin and GJIC in the process of carcinogenesis is associated with a broad spectrum of therapeutic challenges. The modulation of connexin expression and GJIC may be a useful therapeutic method in some types of cancers. However, connexins may both inhibit and facilitate the process of carcinogenesis, in particular metastasis. Therefore, there are different clinical situations in which the inhibition of connexins may be associated with different therapeutic results. The blockade of connexins can be achieved through different mechanisms; therefore, the critical challenge is the search for a mechanism and new drugs selectively modulating the expression of various connexin isoforms.

Additional studies should be performed to explain the mechanisms by which the various connexins regulate the growth, differentiation and invasion of cancer cells. These studies require the use of more specific tools. Further efforts are needed to identify more specific connexin inhibitors such as peptides and peptidomimetics, which have proven very promising. Another critical challenge is to investigate the molecular basis of regulation of different connexin isoforms to explain how the dysregulation of these processes contributes to abnormal cellular connexin location at various stages of cancer progression. Identifying these research challenges will prepare the ground for new diagnostic and therapeutic achievements. It is likely that, with appropriate research including functional studies and bioinformatics, some isoforms of connexins could be found to be markers of particular cancers.

In the coming years, research will focus on the mechanisms regulating the expression of various isoforms of connexins, especially in tumour cells as well as in as well as in other cell types involved in tumour progression such as endothelial cells, fibroblasts, vascular smooth muscle cells and macrophages. Therefore, further studies are needed to explain the role of connexin isoforms in metastasis and angiogenesis in tumours. The critical question is the role of connexins during tumour progression as well as the regulation of connexin expression in tumours. Modulation of connexin expression may be a treatment option in some cancers. Many compounds may decrease or increase the permeability of gap junctions or influence the expression of connexins. Locking certain gap junctions in cancer cells may inhibit their proliferation and growth. Some chemotherapeutics used in clinical practice such as docetaxel may modulate the expression of connexins and may inhibit GJIC [167].

There are several connexin-specific peptides such as Gap19, Gap 24 and Gap27/40 [168]. Unfortunately, their mechanisms of action remain incompletely understood. It is also essential to better understand their pharmacological parameters, side effects, toxicity and mechanisms of anti-tumour action. The challenge is to understand which functions of connexins are most appropriate for therapeutic strategies. Inhibiting gap junctions and hemichannel functions are the most straightforward methods [5]. It has been shown that some peptides such as the Cx43-specific peptide L2 can inhibit hemichannels while simultaneously keeping Cx43 gap junctions in an open state [169]. Thanks to this, we can develop therapeutic strategies for specific connexin functions in a more personalised way. These therapeutic strategies influencing connexin functions may enhance the sensitivity of tumour cells to chemotherapy and increase its effectiveness. By inhibiting connexin function and tumour cell communication, a progressively lower dose of chemotherapy can be used, limiting side effects while maintaining efficacy against cancer cells [170].

Targeting connexins in cancer treatment could be a turning point in cancer management. However, future studies need to concentrate on in situ observations by identifying the precise molecular mechanisms behind the role of Cx in cancer biology, and on designing randomised control trials for particular therapeutic options. For now, it seems that anti-connexin strategies could be more efficient in the treatment of specific tumours rather than globally, as well as in combination treatment with other therapeutic agents rather than alone. Currently, the greatest challenges are to determine the roles of several connexin isoforms in carcinogenesis and metastasis and to continue the search for new selective connexin modulators.

## Figures and Tables

**Table 1:** Clinical relevance of connexins in some types of cancer.

Connexin	Cancer	Clinical Relevance	Reference
Cx46	Breast cancer	Together with extracellular vesicles can be a marker of cancer malignancy in human breast cancer cells; is an independent predictor of the survival of breast cancer patients.	[107,108]
Cx43	Colorectal cancer	Resveratrol may sensitise colorectal cells to cetuximab via upregulating Cx43 to inhibit the Akt pathway in human/mice colorectal cancer cells in vivo and in mice models in vitro; enhances paclitaxel cytotoxicity in human colorectal cancer cell lines.	[109,110]
Cx43	Bladder cancer	Promotion of bladder cancer cell proliferation, migration and invasiveness.	[111]
Cx43	Bone metastases	Bone metastasis exhibited increased expression of Cx43;Cx43 expression in the primary tumour was associated with bone metastasis-free survival.	[112]
Cx43	Lung cancer	Supports malignant progression of non-small cell lung cancer in vivo in human cancer cell lines and in human tumours in vitro	[113]
Cx43	Glioma	Cx43 is expressed in more than 60% of human glioblastoma tissues in different stages.	[114]
Cx43	Melanoma	Dioscin-related upregulation of Cx43 results in decreased migratory and invasive properties of B16 cells and in decreased epithelial–mesenchymal transition in B16 cells and animal tumour tissues.	[115]
Cx32	Hepatocellular carcinoma	Downregulation of Cx32 in hepatocellular carcinoma may be important for HCC cells to acquire epithelial–mesenchymal transition-related acquired drug resistance to oxaliplatin in human cell lines.	[116]
Cx32	Ovarian cancer	Cx32 internalisation by ubiquitin-specific protease 14 inhibition modulates the cisplatin resistance in ovarian cancer cell lines.	[117]

Cx: connexin; HCC: hepatocellular carcinoma.

**Table 2:** Therapeutic strategies targeting connexins in cancer management.

Therapeutic Strategy	Origin	Mechanisms of Action	Clinical Effect	Reference
**Chemical compounds**–**enhancers** Lycopene	Non-provitamin carotenoid derived from various fruits and vegetables	Enhancement of functionality of GJIC; increased Cx43 expression	Inhibitory effect on human breast cancer cell line MCF-7 cell growth	[132]
Lovastatin	Statin; inhibitor of HMG-CoA reductase	Inhibition of PKC; increased GJIC	Reverse in oncogenic Ras-related blockage of GJIC in E9 murine lung carcinoma cells	[134]
Simvastatin	Statin; inhibitor of HMG-CoA reductase	Inhibition of PKC-related phosphorylation of Cx43; increased Cx43 membrane location	Augmentative effect of etoposide in murine Leydig tumour cell line	[135]
Docetaxel	Chemotherapy agent; taxane family member	Induction of apoptosis by downregulation of Bcl-2 and upregulation of caspase-3 activity when combined with Cx43	Antitumor effect in human prostate cancer PC-3 cells in vitro and in vivo	[127]
Cisplatin	Chemotherapy agent	Enhancement of toxicity by suppression of Src activity when combined with Cx43	Increased apoptosis in human mesothelioma H28 cell line	[128]
Sunitinib	Receptor tyrosine kinase inhibitor	Enhanced toxicity in combination with Cx43 through activation of Bax and JNK	Increased chemosensitivity in murine melanoma models in vivo	[140]
EPA	Omega-3 fatty acid	Cx43 upregulation	Increase in apoptosis in human MCF-7 cells in combination with suicide gene therapy in vitro	[141]
All-*trans* retinoic acid	Metabolite of vitamin A1	Cx43 upregulation by promoting transcriptional activation	Sensitises human/mice colorectal cancer cells to cetuximab in vivo and in vitro in mouse models	[157]
Resveratrol	Natural phenol	Cx43 upregulation	Anti-metastatic properties in MDA-MB-231 breast cancer cell line in vitro and in mouse models in vivo	[109]
**Inhibitors** Oleamide	Amide derived from fatty acid oleic acid	Inhibition of gap junctions; blockage of extravasation processes	Inhibition of breast and lung cancer metastasis to the brain; enhancement of cisplatin cytotoxicity	[142]
Tonabersat	Benzopyran derivative; assessed in migraine treatment	Gap junction inhibitor; inhibition of GJ-related cGAMP redistribution	inhibition of breast and lung cancer metastasis to the brain; enhancement of cisplatin cytotoxicity	[91]
Meclofenamate	FDA-approved NSAID drug registered for treatment of joint, muscular pain, arthritis and dysmenorrhea	Gap junction inhibitor; inhibition of GJ-related cGAMP redistribution	Blockage of calcium flow in human breast cancer cells MCF-7	[91]
Carbenoxolone	Glycyrrhetinic acid	GJIC inhibitor	Reduced breast cancer bone metastases in mice ex vivo	[112]
Arsenic trioxide	FDA-approved agent for the treatment of leukaemia	Inhibition of calcium flow via GJ; inhibition of mTOR signalling pathway	Blockage of hemichannels in *Xenopus laevis* oocytes and HeLa cells	[112]
Carbon monoxide	Inhibitor of hemichannels	Unknown	Decreased temozolomide resistance in glioblastoma human cell lines	[144,145,146]
Mimetic peptides αCT1	Synthetic mimetic peptide	Unknown	Enhanced GJ activity and decreased tumour growth in addition to tamoxifen and lapatininb in human breast cancer cells	[148]
TAT-Cx43_266-283_	Mimetic peptide	Blockage of Cx43-ZO-1 interference	Reduced growth, migration, survival of glioma stem cells in patient-derived glioblastoma models	[150]
Gap40	Synthetic peptide	Reduction in stem cells activity by inhibition of Src and FAK	Decreased tumour growth and vascularization in melanoma and papillomavirus oncogene-expressing cells in mice	[151]
**Antibodies** MAbE2Cx43	Anti-Cx43 antibody	Decrease in GJIC due to reduction in Cx40 levels	Tumour reduction in subcutaneous gastric tumours in mice in vivo	[149]
**Viral-carryingtherapy** siRNA	siRNA against Cx37 carried by lentiviruses	Inhibition of the second extracellular loop of Cx43 in the peritumoral invasion zone	Accumulation of antibodies in the peritumoral site of mice gliomas	[152,153]
**Nanocarriers** Cisplatin-loaded nanogel	Synthesised nanogels carrying cisplatin and anti-Cx43 monoclonal antibodies	Reduction in mRNA and protein expression of Cx37		[158]
Liposomal nanocontainers	Synthesised PEGylated liposomal nanocarriers carrying antiCx43 monoclonal antibodies	Targeting Cx43		[155]

GJIC: gap junction intercellular communication; Cx: connexin; HMG-CoA: 3-hydroxy-3-methylglutaryl-coenzyme A; PKC: protein kinase C; Src: proto-oncogene tyrosine-protein kinase; JNK: c-Jun N-terminal kinase; EPA: eicosapentaenoic acids; cGAMP: cyclic guanosine monophosphate-adenosine monophosphate; ZO-1: zonula occludens-1; FAK: focal adhesion kinase; siRNA: small interfering RNA.
